# Students’ perspectives on promoting healthful food choices from campus vending machines: a qualitative interview study

**DOI:** 10.1186/s12889-015-1859-2

**Published:** 2015-05-28

**Authors:** Habiba I. Ali, Amjad H. Jarrar, Mostafa Abo-El-Enen, Mariam Al Shamsi, Huda Al Ashqar

**Affiliations:** Department of Nutrition and Health, College of Food and Agriculture, United Arab Emirates University, PO Box 15551, Al Ain, United Arab Emirates; Faculty of Tourism & Hotels, Hotels Department, University of Alexandria, Alexandria, Egypt

**Keywords:** University students, Vending machines, Nutrition education, Qualitative interviews, United Arab Emirates

## Abstract

**Background:**

Increasing the healthfulness of campus food environments is an important step in promoting healthful food choices among college students. This study explored university students’ suggestions on promoting healthful food choices from campus vending machines. It also examined factors influencing students’ food choices from vending machines.

**Methods:**

Peer-led semi-structured individual interviews were conducted with 43 undergraduate students (33 females and 10 males) recruited from students enrolled in an introductory nutrition course in a large national university in the United Arab Emirates. Interviews were audiotaped, transcribed, and coded to generate themes using N-Vivo software.

**Results:**

Accessibility, peer influence, and busy schedules were the main factors influencing students’ food choices from campus vending machines. Participants expressed the need to improve the nutritional quality of the food items sold in the campus vending machines. Recommendations for students’ nutrition educational activities included placing nutrition tips on or beside the vending machines and using active learning methods, such as competitions on nutrition knowledge.

**Conclusions:**

The results of this study have useful applications in improving the campus food environment and nutrition education opportunities at the university to assist students in making healthful food choices.

## Background

In the United Arab Emirates (UAE), the prevalence of overweight, obesity, and diet-related chronic diseases such as type 2 diabetes has increased dramatically in recent years. This has been attributed mainly to rapid socioeconomic development, which led to changes in eating patterns, food choices, and physical activity levels [1–5]. Increased consumption of energy-dense foods and limited physical activity has resulted in nearly 73 % of adults and 35 % of children and adolescents aged 5–17 being characterized as either overweight or obese [4].

During their college years, students make important decisions about their food choices and these food patterns are likely to be maintained over their lifetime, which influences their future health status. Previous studies have reported that the food choices of university students may not meet current recommendations because of changes associated with moving away from home, busy schedules, and unhealthful eating patterns [6, 7]. Qualitative studies conducted in Europe and the United States reported that a number of individual, social, and environmental factors influenced university students’ food choices [8, 9]. Research conducted in the UAE found a high prevalence of meal skipping and consumption of fast food and soft drinks among college students [10, 11].

Health promotion theoretical frameworks, such as social cognitive theory (SCT) [12] and the social ecological model (SEM) [13], underline the importance of environmental influences on eating behaviors. Briefly, SCT explains the dynamic interactions between personal factors, environmental influences, and behavior. The SEM underscores the multiple environmental factors that influence people’s behavior, including family, peers, school, food outlets, media, culture, social norms, and the political system. More recently, the foodscapes framework has been proposed as a tool with applications in food environment research [14]. The foodscapes framework helps to understand the role of ecological factors, including how the food environment plays a major role in people’s food interactions. This is particularly important in relation to captive eating outside the home environment, such as in schools and universities. The importance of modifying the campus food environment by applying a holistic approach to health promotion in university settings has been highlighted previously [15, 16].

Vending machines are widespread at schools, worksites, and university campuses. Vending machines in schools have been associated with students’ unhealthful food choices [17–20]. A survey of vending machines in 11 college campuses in the United States reported limited healthful food choices [21]. Although college students spend a considerable amount of their time on campus, few studies have been conducted on university vending machines to date [21–26]. Moreover, studies examining factors influencing food choices of university students from campus vending machines in countries undergoing nutrition transition and especially among Arab populations are currently lacking. This issue is particularly important in the UAE because of major shifts to higher energy dietary patterns in recent years [27, 28]. Vending machines are widely available in higher education institutions in the UAE. Vending machines are the most easily accessible food sources in the campus environment. However, the influential factors on the food choices of UAE students at campus-based vending machines are not understood clearly. Moreover, it is important to identify environmental changes and educational interventions aimed at improving university students’ access and selection of healthful foods to enhance their nutritional health.

As part of a larger study investigating the factors influencing students’ food choices from campus vending machines in a major university in the UAE, semi-structured qualitative interviews were conducted with students to determine their opinions on: (1) factors that influence their use of the university vending machines; (2) ways to improve food choices available in the university vending machines; and (3) educational strategies to assist students in making healthier food choices from the campus vending machines.

## Methods

### Setting

This study was conducted in one of the largest universities in the UAE. Student enrollment in 2007/2008 (the year this study was conducted) was approximately 13,000 (78 % female and 22 % male). The majority of students are Emirati citizens, representing all seven emirates (states) of the country. In line with the cultural norms of UAE society, there are separate male and female campuses and vending machines are available within both educational and residential campuses. Depending on their class schedules, students may spend their entire day, from morning to evening, on the educational campus. Students from other cities are provided with on-campus housing by the university. During the period of this study, 146 vending machines were available in the university’s educational and residential campuses. The four major categories of vending machines (according to the foods sold in the machines) in descending order were: snack food machines dispensing items such as potato chips and nuts (35.6 %), coffee and tea machines (24 %), non-diet cola drink machines (17.1 %), and water machines (13.7 %). The remaining categories of vending machines included non-diet non-cola soft drinks, fruit drinks and fruit juice machines (6.9 %), and ice cream and pastry sandwich machines (2.7 %).

### Research design and participants

In-depth individual interviews were conducted with 33 female and 10 male undergraduate students (18–24 years old) enrolled in an introductory nutrition course for non-nutrition majors. They were recruited from students who previously participated in a survey on food choices from university campus vending machines. To achieve the maximum variation of responses [29], students representing various academic colleges of the university, academic years (freshman to senior), living arrangements (on- and off-campus), and backgrounds (i.e., UAE nationals and non-nationals) were recruited. An important aspect of this study was the involvement of students in the various aspects of the research process. Undergraduate students majoring in nutrition were involved in identifying university vending machine improvements as a possible strategy to promote healthful food choices among students. They conducted the interviews and contributed to the study design, pretesting the interview guide, and data analysis. The study protocol was approved by the United Arab Emirates University Research Ethics Committee and participants gave their verbal and written consent.

### Data collection and analysis

In-depth individual interviews were conducted using a semi-structured interview guide. Key questions were related to: 1) students’ perceptions of the university vending machines; 2) factors influencing their food choices from the vending machines; 3) suggestions for improving food items in the campus vending machines; and 4) nutrition education opportunities to improve students’ healthful food choices from campus vending machines. Sample interview questions are given in Table 1. The interview guide was developed by a research team of faculty members and undergraduate students majoring in nutrition. Necessary modifications were made to the interview guide after a pilot test in 11 students (six females and five males). All interviews were conducted during the 2007 fall semester (n = 17) and the 2008 spring semester (n = 26).

**Table 1:** Sample questions included in the qualitative interview guide

1. How do you feel about the availability of vending machines in the university (including the university residential halls)?
- What do you like about them?
- What would you like to change?
2. What are the main reasons you purchase food from the vending machines located at the university? Please explain.
3. What do you think influences your food choices from the vending machines? Please explain.
4. What suggestions do you have to improve the vending machines located in the university?
5. Do you read the labels on vending machine food? Please tell me why or why not.
6. If you are you interested to learn more about how to choose healthy foods from the vending machine, please tell me the ways that you would prefer to learn.

After training in qualitative research techniques, the undergraduate research team members conducted individual interviews in Arabic in a private room on campus. The interviews were audiotaped to keep accurate and complete records of the discussions. Each interview lasted approximately 45–60 min, including the time it took to explain the purpose of the interview and obtain written consent for participation.

The interviews were transcribed verbatim in Arabic. The transcripts were translated into English by the students who conducted the interviews so that the text could be imported into the NVivo software (NVivo 7; QSR International, Doncaster, Australia). This program was used to facilitate thematic analysis and code development of the data. Language equivalency was assured through independent reviews by faculty members fluent in both Arabic and English. In addition, ongoing debriefing among the research team members and references to the original Arabic transcripts were used to verify the translated text.

The transcripts of the interview audiotapes and notes kept during the interviews were analyzed in accordance with qualitative research methods to identify major emerging themes [30]. The constant comparison method [31] was used to identify recurrent patterns and major themes. Categories were systematically compared and grouped into themes as described previously [32]. In line with qualitative research methodology, data collection and analysis were concurrent. After 43 interviews, data saturation was reached as no new themes emerged. Two members of the research team independently analyzed the qualitative data from each semester to improve data trustworthiness [30, 33, 34]. The coding assignments were reviewed and differences were resolved through discussion and consensus by the research team members.

## Results

Table 2 shows the demographic characteristics of the interview participants. Nearly 49 % of the participants lived in the university residential halls while 51 % lived off-campus. The majority of students (30.2 %) were majoring in Business and Economics and the remaining represented the various colleges of the university. Results from the thematic analysis of the in-depth interview transcripts identified four main themes: 1) vending machines are the most easily accessible source of food in the campus; 2) vending machines generally offer low-nutritive food; 3) vending machines need to be improved; and 4) nutrition education should be provided.

**Table 2:** Demographic characteristics of the interview participants (n = 43)

Characteristic	n (%)
Gender	
Female (%)	33 (76.7)
Male (%)	10 (23.3)
Nationality	
UAE (%)	35 (81.4)
Non-UAE (%)	8 (18.6)
Living arrangement	
Off-campus	21 (48.8)
On-campus	22 (51.2)
Academic rank	
Freshman	5 (11.6)
Sophomore	15 (34.9)
Junior	9 (20.9)
Senior	14 (32.6)
Specializations	
Humanities and Social Sciences	8 (18.6)
Business and Economics	13 (30.2)
Food and Agriculture^a^	6 (14.0)
Education	4 (9.3)
Engineering	7 (16.3)
Sciences	5 (11.6)

^a^Nutrition & Dietetics students were excluded

### Vending machines are accessible

A major incentive for students to use the vending machines is their accessibility both on the educational campus and in the university’s residential halls. Participants discussed the importance of having the vending machine in the university educational campus and in the student university residential halls for regular access to food. In addition, some students may not eat breakfast before coming to university because of their early classes and thus use the vending machines. The following statement highlights these findings:

“Of course the vending machines are necessary because sometimes the dormitory cafeteria closes at 10:00 pm and if I feel hungry, I can use the vending machine. Also, in the university, the vending machines are important because sometimes we come to the university very late for the classes and the vending machines are the fastest and nearest place to get food from.” (Female student living on-campus.)

Peer acceptance, busy schedules, and personal preferences influence the university students’ food choices. Female participants indicated that their friends play a major influence on their food choices from the vending machines, as illustrated in the following quotes:

“Friends, definitely friends, because we all just grab something to keep us going, you know … all your classmates are there and you have only 10 minutes before the next class. We all sit around and eat a lot of junk food.” (Female student living on-campus.)

“When my friends buy something, I do too.” (Female student living off-campus.)

“I use the vending machine for quick snacks between lectures; for example, if my classes are in the morning I use the vending machine before lunch. There is little time between classes.” (Male student living on-campus.)

#### Vending machines generally offer low-nutritive food

Some participants were hesitant to purchase food from vending machines. The following quotation highlights this point:

“I do not like the chips and chocolate machine because it contains high-calorie food and they are everywhere; I mean, it encourages students to buy these types of food.” (Female student living on-campus.)

#### Vending machines need to be improved

Most of the participants felt that the food items available in the vending machines at the university should be improved. They made a number of suggestions for ways in which the machine suppliers could improve the nutritional quality of the food items and provide more options to the students.

#### Improve food items offered in the machines

Some participants cited limited healthful food options in the vending machines, whereas others recommended having separate machines for healthful food items and to have products with food labels in the vending machines to facilitate more healthful food choices for the students:

“Increase the healthful food items because some machines rarely have a healthful food product.” (Female student living on-campus.)

“They [vending machine suppliers] can try to bring products with more information on the food label and healthier ones. Also, if we can separate chocolates from the other food, that will be good so that when we go to the vending machines, we would know the healthful ones and I will probably be more inclined to buy healthful food items. If I have everything [junk food] in front of me I might be tempted.” (Female student living off-campus.)

#### Add new machines that include more healthful options

Participants made a number of suggestions for ways in which vending machine food can be improved by adding new machines that provide more healthful food options:

“I encourage the water machine because all of us need a lot of water due to the hot weather. That is why I encourage having this machine everywhere.” (Female student living on-campus.)

“Bring special machines for fresh fruits and vegetables and reduce the sweets and chips.” (Female student living on-campus.)

“Bring vending machines that sell breakfast cereals.” (Female student living off-campus.)

#### Provide nutrition education

In addition to nutrition programs on television, Web sites, and bulletin boards, participants suggested the use of active learning methods, including competitions on nutrition knowledge and food, and nutrition discussions focused on foods sold in the university cafeterias:

“The best way to attract students is to organize lectures on nutrition in the university residential hall cafeteria during dinner and include prizes and competitions among students. In this way it will be more meaningful to us because it is practical.” (Male student living on-campus.)

Other suggested methods of providing nutrition education to students included having nutrition tips and other nutrition information on or beside the vending machines to guide students in making healthful food choices:

“Maybe it will be good to display information related to the topics students need to know about nutrition beside each vending machine because we always forget this information. If this information is available beside the vending machine, we will remember.” (Male student living off-campus.)

“Put tips on vending machine on how to choose healthful foods so that students can read these tips before buying food items … maybe they [students] will change their food choices.” (Female student living off-campus.)

One of the participants was not interested to learn more about nutrition because he felt that he is healthy:

“I will not attend nutrition programs because I have regular medical check-ups and I am healthy. Also, I eat well, but you can prepare these programs [nutrition education] for those who need them.” (Male student living off-campus.)

Participants also discussed some of the reasons they do not read food labels on the food items in the vending machines. These included time constraints, lack of interest in food labels, and lack of skills in food-label reading:

“Yes, the food label is important, but I rarely look at it because I take the product very quickly [from the vending machine] and I do not have time to read the food label. I do not know anything about nutrition; even if there is a food label, I cannot understand it.” (Female student living off-campus.)

## Discussion

The prevalence of obesity in the UAE is a major public health concern requiring preventive programs [4]. In particular, such programs should target food in educational environments because students spend a significant proportion of their time in schools and universities. Both policy and environmental changes are necessary to facilitate healthful food choices. Moreover, it is important to seek university students’ perspectives of their campus food environment. This is appropriate within the foodscapes framework [14] and other ecological models of health promotion, such as the SEM [13] and SCT [12].

We explored students’ suggestions on promoting healthful food choices from campus vending machines as an environmental food change strategy to facilitate healthful food choices among university students in the UAE, a country that has recently undergone nutrition transition [28, 35]. Our study also provides an insight into students’ ideas for providing nutrition education to assist students in choosing more healthful food alternatives from university vending machines. Similar to what has been previously reported for vending machines in high schools [36], universities [21], and health-care institutions [37], the majority of the food items available in this major university’s vending machines were items of low-nutritive value, such as such as potato chips, candy, and non-diet soft drinks. Although there is a food court with a number of fast-food outlets and a convenience store on the campus where this study was conducted, vending machines are a major source of food and water for students because of their convenience, proximity to classrooms, and unlimited hours of operation. Moreover, vending machine food items are not generally more expensive than those sold in the other outlets in the campus which is probably the main reason vending machine food cost was not raised by the study participants.

**Fig. 1:**
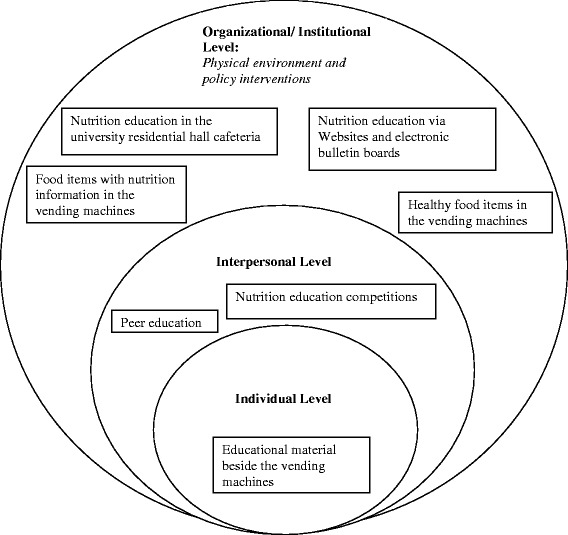
Conceptual framework to improve food choices from campus vending machines

According to the SEM [13], multiple factors in the environment, including schools and food outlets, peers, families, and individual preferences, influence people’s food behaviors. Facilitating behavior changes among college students requires both environmental and individually focused interventions [16, 38]. The participants of this study made suggestions for promoting healthful food choices from campus vending machines in the context of the social ecological theoretical framework for health promotion (Fig. 1).

At the organizational/institutional level, the students suggested environmental and policy changes to the vending machine food to meet their needs, such as increasing the availability of healthful food alternatives and including a nutrition facts panel to assist students in choosing healthful food items. These suggestions would require adoption of additional policies targeting improvements to vending machines as well as working closely with campus vending machine suppliers to decrease the availability of low-nutritive snack choices. Previous research has shown that healthful vending machine policies such as those addressing changes to the product availability and pricing reduced the energy consumed from snacks from vending machines [39, 40]. Moreover, both accessibility and hunger were reported to motivate students to use the vending machines [26], highlighting the importance of food environmental changes. Other organizational-level suggestions included posting nutrition education tips beside the vending machines to prompt greater consideration by students when purchasing food from the vending machines. The effect of vending machine food labeling was found to be more effective when accompanied by an educational poster [25, 41]. Finally, the participants in this study suggested nutrition education via Web sites and electronic bulletin boards, as well as conducting nutrition education activities in the university residential hall cafeteria. It is important to point out that improvements in vending machine food items should be accompanied by nutrition education activities. Provision of more healthful food options in the vending machines alone did not result in an increase in the purchase of healthful food items from the vending machines [26].

At the interpersonal level, study participants mentioned the influence of friends on their vending machine food choices. Educational approaches using peer education and support may be more effective in influencing students’ vending machine food choices. The busy schedules of university students require innovative methods of nutrition education. Moreover, it is important to offer the educational activities in a format that is attractive to young adults, such as competitions involving prizes to promote participation of the students including those who are not generally interested in nutrition.

At the individual level, the students highlighted the need for nutrition education activities to assist them in making healthful food choices from the campus vending machines. Point-of-purchase information can be used as a cost-effective population-based approach to promote healthful food choices among university students [42]. The use of newspapers and emails to promote nutritional education was associated with increased fruit and vegetable consumption among college students [43, 44]. Food label education among college students may reduce consumption of foods high in trans fats [45]. In the US, nutrition labeling of foods/beverages in vending machines was legislated recently [46].

### Strengths and limitations

The qualitative research methodology used in this study as well as the lack of a random process in the selection of the interview participants limits the ability to generalize the findings to university students in the UAE. In addition, students who choose to enroll in a nutrition general education course may be more interested in nutrition and health than the general student population. However, the study recruited non-nutrition majors with diverse backgrounds to seek the maximum variation in responses [29]. Future studies may consider recruiting students not enrolled in nutrition courses to obtain a greater perspective of the students’ opinions. Moreover, although the interview participants represented the various academic colleges in the university, the project conception and implementation involved only students majoring in nutrition. Despite these limitations, to the authors’ knowledge, this is the first study that sought university students’ suggestions on promoting healthful food choices from campus vending machines in the UAE and elsewhere. It is also the first study that identified possible strategies to promote healthful food choices from university vending machines in a country that has recently undergone major food-consumption shifts to more energy-dense dietary patterns. Furthermore, the involvement of students in identifying university vending machine improvements as a possible strategy to promote healthful food choices among students attending this university enhances the relevance of future interventions to meet students’ needs. The use of peer-led interviews to facilitate more candid responses from the participants is also considered an important strength of this study. Finally, suggestions for campus nutrition education strategies are based on the students’ own views.

## Conclusions

The findings of the present study can be useful in developing population-based health promotion activities for university students in the UAE, including nutrition education and modifying the campus food environment. The study may also have implications for nutrition education strategies for university students elsewhere. Future work focusing on implementing students’ recommendations for nutrition education and improving the food items in the university vending machines, as well as evaluating the impact of these changes on students’ food choices is warranted.

## Abbreviations

UAE: United Arab Emirates
SCT: Social Cognitive Theory
SEM: Social Ecological Model
